# Supplementary material for: Initial Treatment with Biological Therapy in Rheumatoid Arthritis
Source: J Clin Med. 2023 Dec 21;13(1):48. doi: 10.3390/jcm13010048 (PMC10779475; doi:10.3390/jcm13010048)
Supplement: Supplementary file 1 [file jcm-13-00048-s001.zip › jcm-2758478-supplementary.pdf]

Supplementary material

Table S1. Search strategies

	**Search term**
	Pubmed
1	"Arthritis, Rheumatoid"[Mesh] OR "rheumatoid arthritis"[Text Word] OR caplan syndrome [tw] OR "caplan's syndrome"[Text Word] OR Caplan Syndromes[tw] OR "rheumatoid nodules"[Text Word] OR "rheumatoid nodule"[Text Word] OR "rheumatoid nodulosis"[Text Word] OR "rheumatoid vasculitis"[Text Word] OR "vasculitis rheumatoid"[Text Word] OR "felty syndrome"[Text Word] OR "felty's syndrome"[Text Word] OR "sjogren's syndrome"[Text Word] OR "sjogrens syndrome"[Text Word] OR "sjogren syndrome"[Text Word] OR "sjoegren syndrome"[Text Word] OR "sjoegren's syndrome"[Text Word] OR "sicca syndrome"[Text Word] OR "still's disease, adult onset"[Text Word] OR "stills disease"[Text Word] OR "adult onset still's disease"[Text Word] OR "adult onset still disease"[Text Word]
2	Abatacept[tw] OR Orencia[tw] OR Adalimumab[tw] OR Humira[tw] OR Baricitinib[tw] OR Canakinumab[tw] OR Certolizumab Pegol[tw] OR Cimzia[tw] OR Clazakizumab[tw] OR Etanercept[tw] OR Enbrel[tw] OR Golimumab[tw] OR Simponi[tw] OR Infliximab[tw] OR Remicade[tw] OR "Interleukin 1 Receptor Antagonist Protein"[Mesh] OR Anakinra[tw] OR Kineret[tw] OR Olokizumab[tw] OR Rituximab[tw] OR Rituxan[tw] OR Sarilumab[tw] OR Secukinumab[tw] OR Cosentyx[tw] OR Sirukumab[tw] OR Tocilizumab[tw] OR actemra[tw] OR biologic disease-modifying anti-rheumatic drugs[tw] OR bDMARDs[tw] OR tumor necrosis factor-alpha[tw] OR TNF-alpha[tw] OR TNF-α[tw] OR tumor necrosis factor[tw] OR biological therapy[tw] OR biological therapies[tw] OR biologic therapy[tw] OR biologic therapies[tw] OR biological factors[tw] OR biological factor[tw] OR biological agents[tw] OR biological agent[tw] OR biological products[tw] OR biological product[tw] OR biologic agent[tw] OR biologic agents[tw] OR biologics[tw]
3	Randomized [All Fields] OR random allocation"[TIAB] NOT Medline[SB]) OR "random allocation"[MH] OR randomized [TW] OR controlled [All Fields] AND ("clinical trials as topic"[MH] OR trial [TW]) OR placebo OR blinded OR randomized controlled trial [Publication Type]
4	Initial [tw] OR Start [tw] OR DMARD-naive OR Methotrexate-naive
	Clinical Queries
1	rheumatoid arthritis and biologic therapy and initial
2	rheumatoid arthritis and biologic therapy and DMARD-naive
3	rheumatoid arthritis and biologic therapy and methotrexate-naive

Table S2. Excluded studies and reasons for exclusion.

#	Study	Reason for exclusion
#	**ADALIMUMAB**	
1	Breedveld_2006 [1]	PREMIER study. The rate of patients with a previous csDMARD was 9-19%
2	Kavanaugh_2013 [2]	OPTIMA study. The rate of patients with a previous csDMARD was 11% and 10% in ADA+MTX and MTX groups
#	**ETANERCEPT**	
3	Bathon_2000 [3]	In the ETN group the rate of patients with a previous csDMARD was almost 20%
4	Emery_2008 [4]	COMET study. The rate of patients with a previous csDMARD was 18% in the ETN+MTX group and 24% in the MTX group
5	Emery_2010 [5]	COMET extension study
6	Kekow_2010 [6]	COMET sub-analysis
#	**INFLIXIMAB**	
7	Rantalaiho_2014 [7]	NEO-RACo study. All patients received a previous csDMARD
8	St Clair_2004 [8]	ASPIRE study. The rate of patients with a previous csDMARD was 29%-35%
9	Tam_2012 [9]	No data regarding previous csDMARDs
#	**TOCILIZUMAB**	
10	BurMter_2016 [10]	FUNCTION study. The rate of patients with a previous csDMARD was 19-23%
11	Jones_2010 [11]	AMBITION study. The rate of patients with a previous csDMARD was 33%-34%
12	Kume_2011 [12]	No data regarding previous csDMARDs
#	**SLR**	
13	Simpson_2019 [13]	The included articles have been already evaluated in our project
14	Singh_2017 [14]	The included articles have been already evaluated in our project
15	Stevenson_2016 [15]	The included articles have been already evaluated in our project

Abbreviations: csDMARD=classical synthetic disease-modifying anti-rheumatic drugs; RCT=randomized controlled trial; ADA=adalimumab; MTX=methotrexate; CZP=certolizumab pegol; ETN=etanercept; SLR=systematic literature review.

Table S3. Evidence table. Main characteristics of included studies.

#	Study	Population	Interventions	Measures	Quality
#	**ADALIMUMAB**				
1	Detert_2013 [16]	- HIT HARD study<br>- Comparisons<br>• ADA+MTX (n=87) vs PBO+MTX (n=85) 24 w<br>• MTX monotherapy up to 48 w<br>- Population<br>• Woman [n (%)]: 61 (70.1%) vs 57 (67.1%); p=0.67<br>• Age (yr): 47.2±12.1 vs 52.5±14.3; p=0.009<br>• Disease duration (m): 1.8±2.1 vs 1.6±1.7; p=0.57<br>- Clinical characteristics<br>• RF+ n (%): 55 (63.2%) vs 59 (69.4%); p=0.51<br>• DAS28: 6.2±0.8 vs 6.3±0.9; p=0.60<br>• TJC 0–28: 13.0±6.5 vs 13.1±5.9; p=0.61<br>• SJC 0–28: 10.2±5.0 10.7±4.5; p=0.61<br>• TJC 0–68: 20.9±12 vs 19.4±10.6; p=0.38<br>• SJC 0–66: 13.6±7.1 vs 14.0±6.9; p=0.76<br>• HAQ-DI 1–3: 1.4±0.61 vs 1.3±0.62; p=0.49<br>• SF-36 mental: 46.7±9.9 vs 45.2±10.2; p=0.34<br>• SF-36 physical: 28.3±7.7 vs 31.7±8.3; p=0.010<br>- Structural damage: SHS<br>• Total 0–448: 6.3±5.0 vs 11.4±14.8; p=0.19<br>• Erosions 0–280: 2.2±3.0 vs 4.4±8.7; p=0.62<br>• Joint space narrowing 0–168: 4.1±3.6 7.0±7.3; p=0.047	- MTX (15 mg/w) monotherapy vs MTX + ADA<br>• ADA (40 mg) or PBO sc / 2 w for 24 w<br>• MTX monotherapy in both groups up to 48 w<br><br>- All patients: folic acid (10 mg/w)<br>- Prednisone ≤10 mg/d allowed	- Efficacy:<br>• DAS28 48 w<br>• DAS28 24 w<br>• Remission (DAS28 <2.6)<br>• ACR 20/50/70<br>• TJC 0–28<br>• SJC 0–28<br>• HAQ-DI<br>• Δ SHS<br><br>- Safety<br>• AE and severe AE	4
2	Soubrier_2009 [17]	- GUEPARD study: MTX monotherapy vs MTX + ADA<br>- Population<br>• Woman [n (%)]: 26 (81.2%) vs 26 (78.8%); p=1.00<br>• Age (yr): 49.3±15.2 vs 46.3±16.3; p= 0.44<br>• Disease duration (m): [4.4 (3.3–5.1)] vs [4.4 (2.5–5.4)]; p=0.54<br>- Clinical characteristics	- MTX monotherapy vs MTX + ADA.<br>- Every 3 m medications adjustments if DAS≤3.2<br><br>- MTX: 0.3 mg/kg/w then up to 20 mg/kg/w<br>• If remission ↓ doses (2.5 mg/m) up to 7.5 mg/w	- Efficacy<br>• Low activity (w 12)<br>• Time to low activity<br>• Anti-TNF-α doses<br>• Morning stiffness, pain, Physician VAS, fatigue, ESR	3

		• TJC: 14.1±6.4 vs 13.8±7.1; p= 0.87<br>• SJC: 10.8±5.3 vs 9.5±4.5; p=0.26<br>• ESR: 35.1±23.8 vs 39.4±20.9; p=0.45<br>• DAS28-ESR: 6.15±0.88 vs 6.31±0.7; p=0.44<br>• HAQ 0–3: 1.41±0.74 vs 1.69±0.59; p= 0.10<br>- Structural damage: SHS<br>• Total 0–448: 7.5±21.3 vs 2.4±4.6; p= 0.19<br>• Erosions 0–280: 3.9±12.4 vs 1.2±2.1; p= 0.21<br>• Joint space narrowing 0–168: 3.6±9.2 vs 1.3±3.2; p=0.19<br>• Erosions hands and feet n (%): 12 (37.5) vs 10 (31.25); p= 0.79	- ADA: 40 mg/ 2 w<br>• If DAS28>3.2 stop ADA<br><br>- Prednisone ≤10 mg/d allowed	• ACR20/50/70<br>• EULAR response<br>• HAQ (0–3)<br>• SF-36<br><br>- Safety<br>• AE and severe AE	
3	Hørslev-Petersen_2014 [18]	- OPERA study: ADA+MTX (n=89) vs PBO+MTX (n=91)<br>- Population<br>• Woman (%): 63% vs 69%; p=0.46<br>• Age (yr): 56.2 (25.8–77.6) vs 54.2 (28.3–76.7); p=0.71<br>• Disease duration (d): 88 (42–162) vs 83 (42–150); p=0.74<br>• RF+ (%): 70% vs 74%; p=0.67<br>• TJC (0–40): 15 (5–38) vs 16 (6–34); p=0.78<br>• SJC (0–40): 10 (3–33) vs 11 (3–31); p=0.66<br>• DAS28-ESR: 5.5 (3.8–7.8) vs 5.6 (3.8–7.3); p=0.53	- MTX: 7.5 mg/w oral, ↑ to 15 mg/w at 1 m, and up to 20 mg/w at 2 m<br>- ADA: 40 mg/ 2 w<br><br>- Intra-articular triamcinolone (4 ml) in swollen joints (maximum 4) at baseline and subsequent visits allowed	- Efficacy at 12 m<br>• Low disease activity (DAS28-CRP≤3.2)<br>• Remission (DAS28-CRP≤2.6, CDAI≤2.8, SDAI≤3.3)<br>• ACR/EULAR 28/40<br>• HAQ<br>• SF-36	3
#	**CERTOLIZUMAB PEGOL**				
4	Emery_2017 [19]	- C-EARLY study: PBO+MTX (n=213) vs CZP+MTX (n=665)<br>- Population<br>• Age (yr): 51.2±13.0 vs 50.4±13.6<br>• Woman n (%): 170 (79.8%) vs 497 (75.9%)<br>• Disease duration (m): 2.9±2.9 vs 2.9±4.6<br>• DAS28-ESR: 6.8±0.9 vs 6.7±0.9<br>• CDAI: 42.6±12.9 vs 41.3±12.5<br>• HAQ-DI: 1.7±0.6 vs 1.6±0.6<br>• TJC (28): 16.2±6.5 vs 15.6±6.5<br>• SJC (28): 13.0±5.6 vs 12.4±5.5<br>• ESR [median (min, max)]:[44.0 (10.0-135.0)] vs [42.0 (2.0-150.0)<br>• RF+ (≥14 IU/mL) n(%): 206 (96.7) vs 634 (96.8)	- PBO+MTX vs CZP+MTX<br><br>- CZP: 400 mg sc 0, 2 and 4 w, then 200 mg/2 w to w 52<br>- MTX: 10 mg/w, ↑ doses (5 mg/2 w) up to 25 mg/w.	- Efficacy: 40 and 52 w<br>• Remission (DAS28-ESR<2.6)<br>• Low disease activity (DAS-ESR≤3.2)<br>- Efficacy: 52 w<br>• ACR50<br>• Δ HAQ-DI y HAQ-DI≤0.5 (%)<br>• Δ mTSS<br>• Δ DAS-ESR<br>- Efficacy: 12, 24, and 52 w<br>• DAS28-ESR <2.6	4

		• mTSS. [median (min. max)]: 2.8 (0. 161) vs 3.0 (0. 130)<br>• mTSS: 8.5±17.5 vs 7.2±13.8<br>• Joint space narrowing [median (min, max)]: 0 (0. 94) vs 0 (0. 76)<br>• Erosions. n (%): 169 (79.3%) vs 506 (77.3%)		• CDAI≤2.8<br>• SDAI≤3.3<br><br>- Safety: AE and serious AE	
5	Hetland_2020 [20]	- MTX combined therapy:<br>• Conventional treatment (CT): (prednisone or SFZ+CLQ and intraarticular steroids)<br>• CZP<br>• ABT<br>• TCZ<br>- (CT+MTX) vs (CZP+MTX) vs (ABT+MTX) vs (TCZ+MTX)<br>- Population (n=812)<br>• Age (yr): (54.6±14.5) vs (55.3±15.3) vs (54.7±14.4) vs (52.4±14.5)<br>• Woman n(%): 139 (69.5) vs 139 (68.5) vs 140 (68.6) vs 129 (68.6)<br>• Disease duration (d): (195±167) vs (203±166) vs (212±168) vs (208 ±155)<br>• RF+ n (%): 151 (75.5) vs 149 (73.4) vs 159 (77.9) vs 135 (71.8)<br>• CDAI: (28.6±12.1) vs (27.9±12.4) vs (28.6±11.3) vs (26.6±11.7)<br>• DAS28-CRP: (5.1±1.1) vs (5.0±1.1) vs (5.1±1) vs (4.9±1)<br>• TJC (68 art): (17±11.4) vs (15.3±10.4) vs (16.1±10.7) vs (14.8±10.2)<br>• SJC (66 art): (11.4±7.3) vs (11.2±7.6) vs (11.1±7.3) vs (9.8±6.4)<br>• HAQ (0-3): (1.1±0.6) vs (1±0.6) vs (1.1±0.6) vs (1.1±0.5)	-MTX: Doses escalation/4 w up to 25 mg/w<br>- MTX + CT: prednisone and ↓ doses up to 5-20 mg/d in 9 w or SFZ (2 g/d) + HCQ (35 mg/kg/w or 200 mg/d) and intra-articular steroids at each visit)<br>- MTX + CZP 400 mg sc 0, 2 and 4 w, then 200 mg/2 w<br>- MTX + ABT (125 mg/w sc)<br>- MTX + TCZ (8 mg/kg/4 w iv or 162 mg/w sc)	- Efficacy: 2, 4, 8, 12, 16 and 24 w<br>• Remission CDAI (≤2.8), SDAI (≤3.3), DAS28-ESR (<2.6), ACR/EULAR 12, 24 w<br><br>- Safety: AE and serious AE	3
6	Weinblatt_2017 [21]	- C-EARLY study (CZP_Emery_2017): CZP + MTX<br>- Low disease activity w 40 and 52 (n=293):<br>• CZP standard dose (n=84): CZP (200 mg/2 w) + MTX<br>• CZP optimization (n=127): CZP (200 mg/4 w) + MTX<br>• CZP stop (n=82): PBO/2 w + MTX	- CZP standard dose (200 mg/2 w + MTZ)<br>- CZP optimization (200 mg/4 w + MTZ)<br>- CZP stop (PBO/2 w + MTX)	- Efficacy: w 104<br>• Low disease activity (DAS-ESR≤3.2)<br>• Remission (DAS<2.6)	5

		Population (C-EARLY study):<br>CZP (standard dose) vs CPZ (optimization) vs CZP (stop) vs MTX responders<br>- DAS28-ESR:<br>• Basal: (6.4±1.0) vs (6.6±0.8) vs (6.5±0.8) vs (6.6±0.9)<br>• W 52: (2.0±0.7) vs (2.0±0.6) vs (1.9±0.7) vs (2.2±0.7)<br>- SJC (28 art.):<br>• Basal: (11.3±5.3) vs (12.4±5.1) vs (11.3±4.8) vs (11.9±4.8)<br>• W 52: (0.3±1.2) vs (0.5±1.4) vs (0.3±0.7) vs (0.7±1.6)<br><br>- HAQ-DI:<br>• Basal: (1.6±0.6) vs (1.6±0.6) vs (1.5±0.5) vs (1.5±0.6)<br>• W 52: (0.3±0.4) vs (0.3±0.5) vs (0.3±0.5) vs (0.4±0.5)<br>- SHS:<br>• Basal: (3.1±5.4) vs (4.5±9.2) vs (5.1±8.5) vs (6.0±12.4)<br>• W 52: (3.3±5.1) vs (4.5±8.9) vs (5.0±7.4) vs (6.8±12.7)	- MTX: 10 mg/w and dose escalation 5 mg/ 2 w up to25 mg/w (minimum 15 mg/w). This dose during 52-103 w<br><br>-CZP: 200 mg/w 52-103	• No radiographic progression (Δ SHS≤0.5)<br>• HAQ-DI ≤0.5<br><br>- Safety: AE and serious AE	
#	**ETANERCEPT**				
7	Nam_2014 [22]	- EMPIRE study: MTX+PBO (n=55) vs MTX+ETN (n=55)<br>- Early synovitis (≥1 swollen and painful joint)<br>- Population: MTX+PBO vs MTX+ETN<br>• Age (yr): (48.4±13.3) vs (47.9±13.6)<br>• Woman n (%): 40 (72.7%) vs 44 (80.0%)<br>• Disease duration (m): [8 (6 a 11)] vs [6 (4 a 9)]<br>• DAS44-CRP: (2.95±0.91) vs (2.94±0.92)<br>• DAS28-CRP: (4.17±1.10) vs (4.10±1.14)<br>• RF+ n (%): 30 (55.6%) vs 31 (56.4%)<br>• HAQ-DI: (1.00±0.43) vs (1.01±0.47)<br>• SF-36 mental: (42.45±12.23) vs (46.82±10.19)<br>• SF-36 physical: (35.51±7.90) vs (36.00±8.04)<br>• EQ-5D-3L: (0.569±0.252) vs (0.578±0.245)<br>• Erosions: (1.36±2.95) vs (1.10±1.84)<br>• Joint space narrowing: (6.65±6.03) vs (5.59±4.28)<br>• mTSS: (8.01±8.06) vs 6.69±5.04)	-ETN: 50 mg/w sc<br><br>-MTX: 10 mg/w y ↑ (5 mg/4 w) up to 25 mg or maximum tolerate dose. If treatment objective MTX 15 mg/w<br><br>- Intra-articular or intra-muscular steroids allowed (maximum metilprednisolone dose 120 mg)	- Efficacy:<br>52 w:<br>• No pain/swollen joints<br><br>78 w:<br>• VAS pain, fatigue<br>• DAS44-CRP<br>• Remission ACR/EULAR<br>• HAQ-DI<br>• EQ-5D 3L<br>• SF-36<br>• Δ mTSS<br><br>-Exploratory resuts:<br>• DAS28-CRP<br>• HAQ normal (≤0.5)	5

				- Safety: AE and serious AE	
#	**INFLIXIMAB**				
8	Bejarano_2010 [23]	IFX+MTX vs MTX<br>- Population (n=20)<br>• Age (yr): [51 (41-55)] vs [46 (40-63)]; p=0.83<br>• Woman n (%): 6 (67%) vs 6 (67%)<br>• Disease duration (m): [6 (3-12)] vs [5 (3-11)]; p=0.82<br>• RF+ (%): 6 (67%) vs 6 (67%)<br>• CRP: [48 (15-66)] vs [20 (11-55)]; p=0.33<br>• DAS-28: [6.3 (5.6-6.5)] vs [6.9 (6.1-7.9)]; p=0.09<br>• HAQ 0-3: [1.3 (0.8-1.6)] vs [1.4 (1.1-1.9)]; p=0.66<br>• RAQoL: [11 (9-21)] vs [23(14-26)]; p=0.04	-IFX (3 mg/kg) or PBO iv during 1 yr: 0, 2, 6 w and then every 8 w<br>-All patients with MTX (monotherapy)	- Efficacy (8 yr):<br>W 52:<br>• HAQ<br>• RAQoL<br>• Remission (DAS28)<br>• Remission without treatment	2
9	Durez_2007 [24]	MTX vs MP-IV vs IFX<br>Population (n=44): MTX (n=14) vs MTX+MP-IV (n=15) vs MTX+IFX (n=15)<br>• Active disease (SJC≥6 and TJC≥8)<br>• Woman: 71% vs 60% vs 67%<br>• Age (yr): 53.8±15.2 vs 50.3±14.2 vs 50.0±9.9<br>• Disease duration (yr): 0.45±0.29 vs 0.25±0.33 vs 0.36±0.31<br>• RF+: 64% vs 100% vs 67%<br>• Anti-CCP+: 42% vs 92% vs 73%<br>• DAS28-CRP: (5.2±0.8) vs (5.3±1.3) vs (5.3±1.1)<br>• SJC: (10.3±5.5) vs (12.4±7.6) vs (12.5±5.4)<br>• TJC: (11.6±7.5) vs (13.2±9.1) vs (15.9±8.0)<br>• HAQ: (1.3±0.6) vs (1.2±0.7) vs (1.5±0.8)<br>• Radiographic erosions: 36% vs 33% vs 13%	-All patients MTX: 7.5 mg/w then 20 mg/w at 14 w<br>-MP-IV: 1 g<br>-IFX: 3 mg/kg w 0-2-6 then every 9 w up to w 46	- Efficacy:<br>Basal-2-6-14-22-30- 38 w<br>• MRI: synovitis, edema, erosion<br>W 22 and 52<br>• DAS28- CRP<br>• ACR20/50/70<br>• HAQ<br><br>- Safety: Serious AE	2
10	Goekoop-Ruiterman_2005 [25]	- BeSt study (1st yr)<br>- csDMARD monotherapy sequential vs combined therapy sequential vs combination with prednisone vs combination with IFX<br>- Population (n=508): 126 vs 121 vs 133 vs 128<br>• Age (yr): (54±13) vs (54±13) vs (55±14) vs (54±14)<br>• Woman n (%): 86 (68%) vs 86 (71%) vs 86 (65%) vs 85 (66%)	1.csDMARD monotherapy sequential<br>• MTX (15 mg/w and↑ 25-30 mg/w if DAS44>2.4). Low response (DAS44>2.4): SSZ, LFN, MTX+IFX combination sequential. Therapeutic failure: change to MTX+IFX; MTX+CSA+prednisone<br>2.Combination with prednisone	- Efficacy 12 m (every 3 m)<br>• D-HAQ<br>• Radiographic progression: SHS, erosions, joint space narrowing	3

		• Disease duration (w): [23 (14–54)] vs [26 (14–56)] vs [23 (15–53)] vs [23 (13–46)]<br>• RF+: 84 (67%) vs 77 (64%) vs 86 (65%) vs 82 (64%)<br>• DAS44: (4.5±0.9) vs (4.5±0.8) vs (4.4±0.9) vs (4.3±0.9)<br>• D-HAQ (0-3): (1.4±0.7) vs (1.4±0.6) vs (1.4±0.7) vs (1.4±0.7)<br>• SHS (0-448): [3.5(1.5-9.5)] vs [5.0(1.5-8.1)] vs [3.5(1.5-8.5)] vs [4.0(1.5-8.5)]<br>• Erosion (0-280): 4.1±6.2 vs 3.5±4.3 vs 3.3±4.3 vs 3.9± 5.8<br>• Joint space narrowing (0-168): 3.2±4.9 vs 2.8±3.2 vs 2.6±3.2 vs 3.1±5.2<br>• Radiographic erosions hands and feet n (%): 89(72%) vs 82(70%) vs 93(71%) vs 93(73%)	• MTX (7.5 mg/w), SSZ (2000 mg/d) and prednisone (60 mg/d)<br>• No response: MTX ↑ 25-30 mg/w; addition CSA and prednisone, IFX, LFN, MP, AZA<br>3. Combination with IFX<br>• MTX (25-30 mg/w)+IFX (3 mg/kg; w 0-2-6 and then every 8 w)<br>• At 3 m ↑ IFX to 6 mg/kg/ 8 w if DAS44 >2.4	- Safety: AE and serious AE	
11	Goekoop-Ruiterman_2007 [26]	- BeSt study (2nd yr)<br>- Population (n=508): 126 vs 121 vs 133 vs 128<br>• Age (yr): (54±13) vs (54±13) vs (55±14) vs (54±14)<br>• Woman n (%): 86 (68%) vs 86 (71%) vs 86 (65%) vs 85 (66%)<br>• Disease duration (w): [23 (14–54)] vs [26 (14–56)] vs [23 (15–53)] vs [23 (13–46)]<br>• RF+: 84 (67%) vs 77 (64%) vs 86 (65%) vs 82 (64%)<br>• DAS44: (4.5±0.9) vs (4.5±0.8) vs (4.4±0.9) vs (4.3±0.9)<br>• D-HAQ (0-3): (1.4±0.7) vs (1.4±0.6) vs (1.4±0.7) vs (1.4±0.7)<br>• SHS (0-448): [3.5(1.5-9.5)] vs [5.0(1.5-8.1)] vs [3.5(1.5-8.5)] vs [4.0(1.5-8.5)]<br>• Erosion (0-280): 4.1±6.2 vs 3.5±4.3 vs 3.3±4.3 vs 3.9± 5.8<br>• Joint space narrowing (0-168): 3.2±4.9 vs 2.8±3.2 vs 2.6±3.2 vs 3.1±5.2<br>• Radiographic erosions hands and feet n (%): 89(72%) vs 82(70%) vs 93(71%) vs 93(73%)	4.csDMARD monotherapy sequential<br>• MTX (15 mg/w and↑ 25-30 mg/w if DAS44>2.4). Low response (DAS44>2.4): SSZ, LFN, MTX+IFX combination sequential. Therapeutic failure: change to MTX+IFX; MTX+CSA+prednisone<br>5.Combination with prednisone<br>• MTX (7.5 mg/w), SSZ (2000 mg/d) and prednisone (60 mg/d)<br>• No response: MTX ↑ 25-30 mg/w; addition CSA and prednisone, IFX, LFN, MP, AZA<br>6. Combination with IFX<br>• MTX (25-30 mg/w)+IFX (3 mg/kg; w 0-2-6 and then every 8 w)<br>At 3 m ↑ IFX to 6 mg/kg/ 8 w if DAS44 >2.4	- Efficacy:<br>• D-HAQ<br>• Radiographic progression: SHS, erosions, joint space narrowing<br><br>- Safety: AE and serious AE	3
12	Goekoop-Ruiterman_2008 [27]	Best study (1st yr), see Goekoop-Ruiterman_2005	See Goekoop-Ruiterman_2005	See Goekoop-Ruiterman_2005	3
13	Markusse_2016 [28]	Best study (10 yr), see Goekoop-Ruiterman_2005	See Goekoop-Ruiterman_2005	See Goekoop-Ruiterman_2005	3

14	Nam_2014 [29]	- IDEA study: IFX+MTX vs MP-IV+MTX<br><br>- Population (n=112)<br>• Age (yr): 52.9±12.8 vs 53.7±13.0<br>• Woman n (%): 41(71.9%) vs 36 (65.5%)<br>• Disease duration (m): [1.2 (0.7. 2.1)] vs [1.2 (0.7. 1.7)]<br>• CRP (mg/ml): [18 (10. 51)] vs [16 (7.61)]<br>• DAS44: 3.56±0.98 vs 4.05±1.04<br>• RF+ n (%): 34 (60.7%) vs 27 (49.1%)<br>• HAQ-DI: 1.339±0.539 vs 1.426±0.527<br>• Erosions: 3.51±8.73 vs 1.36±2.91<br>• Joint space narrowing: 5.72±9.93 vs 4.69±8.38<br>• mTSS: 9.23±18.31 vs 6.05±10.83	- MTX+IFX (3 mg/kg) to w 26:<br>• DAS44≤2.4: same treatment<br>• DAS>2.4: ↑ IFX doses and then change csDMARD<br><br>- MTX+MP-IV (250 mg) then PBO<br>• DAS44≤2.4: same treatment<br>• DAS>2.4: csDMARD escalation	- Efficacy:<br>Radiographic progression<br>• Δ mTSS (w 50)<br>• Δ Erosions<br>• Δ Joint space narrowing<br><br>Remission<br>• DAS44<1.6<br>• During 6 m<br><br>PROs<br>• HAQ-DI y RAQoL<br><br>- Safety: AE and serious AE	4
15	Quinn_2005 [30]	IFX+MTX vs PBO+MTX<br><br>- Population (n=20)<br>• Age (yr): 51.3±9.5 vs 53.1±13.7<br>• Disease duration (m): 7.4±4.6 vs 6±3.7<br>• RF+: 70% vs 60%<br>• CRP (mg/l): 47±27.9 vs 37±38.8<br>• HAQ: [1.3 (0.875)] vs [1.3 (0.97)]	- IFX (3 mg/kg): 0, 2, 6 and every 8 w up to w 46<br><br>-MTX (7.5 mg/w) + folic acid (5 mg 2/w) and ↑ gradual dose escalation	Efficacy 14 and 52 w:<br>• MRI: synovitis, edema, erosion<br>• Radiographic progression: SHS<br>• DAS28<br>• ACR20/50/70 response<br>• HAQ and RAQoL	3
#	**TOCILIZUMAB**				
16	Bijlsma_2016 [31]	U-Act-Early study: TCZ+MTX (n=106) vs TCZ (n=103) vs MTX (n=108).<br><br>- Population (n=317):<br>• Woman: 61% vs 76% vs 64%<br>• Age (yr) [53.0 (46-60)] vs [55 (47-63)] vs [53.5 (44.5-62)]<br>• RF+: 75% vs 75% vs 86%<br>• Disease duration (d): [24.5 (16-45.5)] vs [25.5 (18-45)] vs [27 (15-46)]<br>• DAS28 median: 5.2 (1.1) vs 5.3 (1.1) vs 5.1 (1.2)<br>• TJC44 median: 10 (7-17) vs 11 (7-18). vs 10 (5.5-17)	• TCZ: 8 mg/kg/4 w iv (maximum 800 mg/doses)<br>• MTX: 10 mg/w oral + folic acid and ↑ 5 mg/4 w up to 30 mg/w<br><br>- If no remission with maximum MTX dose: addition HCQ 200 mg 2/d during 3 m	- Efficacy 2 yr<br>Remission:<br>• DAS28<2.6 and SJC≤4<br>• Maintained at least 24 w<br><br>• EULAR response<br>• ACR20/50/70/90<br>• HAQ<br>• Radiographic progression	4

		• SJC44 median: 9 (6-15) vs 11 (7-18) vs 10 (5.5-17)<br>• ESR (mm/h): 23.5 vs 27.0 vs 25.0<br>• CRP (mg/l): 8.0 vs 9.0 vs 9.6			
17	Verhoeven_2020 [32]	U-Act-Early study (3rd yr)<br>n=226 (71% of initial study)<br><br>TCZ+MTX (n=75) vs TCZ (n=79) vs MTX (72)<br>• Woman: 63% vs 77% vs 67%<br>• RF+: 71% vs 71% vs 82%<br>• Age: 53.8±11.2 vs 55.5±11.6 vs 53.7±12.9<br>• Disease duration (d): [27 (18-43)] vs [25 (19-43)] vs [28 (16-46)]<br>• DAS28: 5.1±1.1 vs 5.3±1.1 vs 5.0±1.2<br>• HAQ: [1.3 (0.6-1.6)] vs [1.3 (0.9-1.8)] vs [1.0 (0.5-1.4)]	Treatment during 2 yr:<br>• TCZ: 8 mg/kg/4 w iv (maximum 800 mg/doses)<br>• MTX: 10 mg/w oral + folic acid and ↑ 5 mg/4 w up to 30 mg/w<br><br>If maintained remission MTX dose reduction until interruption, then TCZ dose reduction until interruption	- Efficacy 3 yr<br><br>• DAS28<2.6 and SJC≤4<br>• EULAR response<br>• ACR20/50/70/90<br>• HAQ<br>• Radiographic progression	4

Abbreviations: ABT=abatacept; ACR=American College Rheumatology; ADA=adalimumab; CDAI=Clinical Disease Activity Index; HAQ=health assessment questionnaire; D-HAQ=Dutch version of HAQ; mTSS= van der Heijde modified total Sharpe score; MP= metilprednisolone; RAQoL=Rheumatoid Arthritis Quality of Life Scale; SDAI=Simplified Disease Activity Index; SHS= Sharp-van der Heijde index; SFZ=sulphasalazine; TCZ=Tocilizumab; TJC=tender joint count; SJC=tender joint count; m=month; yr=year; d=day; AE=adverse event; sc=subcutaneous; iv=intravenous; ESR=erythrocyte sedimentation rate; VAS visual analogic scale; CRP=C reactive protein; MRI= magnetic resonance imaging.

Tabla S4. Main results of included studies.

Study	Efficacy	Safety	Main conclusion
**ADALIMUMAB**			
Detert 2013 [16]	ADA/MTX vs PBO/MTX: W 48<br>• DAS28: 3.2±1.4 vs 3.4±1.6; p= 0.41<br>• TJC (0–28): 3.4±5.1 5 vs 5.0±6.5; p= 0.080<br>• SJC (0-28): 1.5±2.4 vs 2.9±4.3; p=0.011<br>• ESR (mm): 19.5±15.0 vs 18.6±15.3; p=0.37<br>• Remission DAS28: 42.4% vs 36.8%; p=0.47<br>• Response:<br>o ACR20 (%):66.0 vs 74.9; p=0.21<br>o ACR50 (%):52.6 vs 51.4; p=0.88<br>o ACR70 (%): 40.5 vs 34.0; p=0.40<br>• HAQ-DI (1-3): 0.61±0.6 vs 0.66±0.6; p=0.40	ADA/MTX (n=87) vs PBO/MTX (n=85)<br>Serious AE: 12 (13.7%) vs 22 (19.5%)<br>Total serious infections 3 vs 4<br>Total cardiac events: 1 vs 3<br>Total psychiatric events 0 vs 1<br>Reactive depression 1 vs 0<br>Cancer 0 vs 3<br>Total gastrointestinal events 1 vs 0<br>Total vascular events 1 vs 1<br>Total autoimmune events 1 vs 0	A greater reduction in radiographic progression after initial combination therapy with ADA and MTX was seen at week 48, even after discontinuation of ADA treatment at week 24. This sustained effect was not found at the primary endpoint (DAS28 reduction).
Soubrier 2009 [17]	MTX vs MTX+ADA<br>Low disease activity (DAS) (n): 7 vs 11 (p=0.28)<br>Time to low disease activity: 19.6 (16.9-26.0) vs 12.0 (8.3-12.6) w; p=0.017<br>Nº of visits with low disease activity: 10.4 (7.6-13.3) vs 13.4 (10.6-16.2); p=0.11<br><br>W 12: MTX+ADA vs MTX<br>• Improvement in morning stiffness: p=0.028<br>• Improvement VAS physician: p=0.0017<br>• No differences in pain (p=0.19), fatigue (p=0.20), ESR (p=0.12)<br><br>W 12: MTX vs MTX+ADA:<br>• ACR20: 50% vs 84%; p<0.05<br>• ACR50: 27% vs 66%; p<0.05<br>• ACR70: 19% vs 44%; p<0.05<br>• EULAR (good response: DAS <3.2 y ↓punt basal >1.2): 25% vs 63.6%; p=0.0014<br>• EULAR (low activity: DAS<3.2): 25% vs 63.6%; p=0.0014<br>• EULAR (remission DAS<2.6): 12.5 vs 36.4; p=0.0223	MTX+ADA vs MTX<br><br>Cancers: 2 vs 0<br>Serious AE 3 vs 0<br>Hospitalizations 5 vs 0	Initial combination of MTX and ADA and then an adjusted based on the disease activity status achieved a faster control of disease activity but did not increase the number of patients for whom anti-TNF-_ treatment was not needed after 12 weeks nor a better subsequent clinical or radiological outcome than a 3-month delayed initiation of anti-TNF in patients with still active disease despite MTX.

	W 52 MTX vs MTX+ADA:<br>• ACR20: 81% vs 85%; p>0.05<br>• ACR50: 68% vs 67%; p>0.05<br>• ACR70: 42 vs 58%; p>0.05<br>• EULAR (good response: DAS <3.2 y ↓punt basal >1.2): 65.6% vs 63.6%; p=0.98<br>• EULAR (low activity: DAS<3.2): 65.6% vs 63.6%; p=0.98<br>• EULAR (remission DAS<2.6): 59.4% vs 39.4%; p=0.15<br><br>HAQ: MTX vs MTX+ADA (95% CI)<br>• W 12: -0.51; (-0.30.-0.72) vs -0.82 (-0.52. -1.11), p=0.26<br>• W 52: -0.93 (-0.69. -1.17) vs -1.02 (-0.81. -1.24), p=0.79<br>No differences in radiographic progression (p=0.18), SF-36 at 1 yr		
Hørslev-Petersen 2014 [18]	<u>ADA+MTX vs PBO+MTX</u><br>Year 1<br>• TJC (0–28): 0 (0–13) vs 0 (0–9); p=0.08<br>• SJC (0–28): 0 (0–6) vs 0 (0–3); p=0.20<br>• CRP (7–161): 7 (7–21) vs 7 (7–44); p=0.21<br>• DAS28-CRP (1.7–8.7): 2.0 (1.7–5.2) vs 2.6 (1.7–4.7); p=0.009<br>• CDAI (0–76): 1.9 (0–27.9) vs 3.9 (0.1–17.2); p=0.01<br>• SDAI (0.7–82): 2.7 (0.7–30.4) vs 5.0 (0..8–20.2); p=0.006<br>• DAS28-ESR<3.2: 80% vs 76. p=0.65<br><br>Remission<br>• DAS28-CRP<2.6: 74% vs 49%; p=0.0008<br>• CDAI≤2.8: 61% vs 41%; p=0.008<br>• SDAI<3.3: 57% vs 37%; p=0.007<br>• ACR/EULAR 28: 48% vs 30%; p=0.014<br>• ACR/EULAR 40: 47% vs 26%; p=0.005<br><br>Response<br>• ACR20 86% vs 78%; p=0.21<br>• ACR50 80% vs 63%; p=0.020<br>• ACR70 65% vs 45%; p=0.012	-	Adalimumab added to methotrexate and intra-articular triamcinolone as first-line treatment did not increase the proportion of patients who reached the DAS28CRP<3.2 treatment target, but improved DAS28CRP, remission rates, function and quality of life in DMARD-naïve ERA.

	Function and quality of life: Δ 0-12 m<br>• HAQ (0-3): -0.88 (-2.46; -0.13) vs -0.63 (-1.82; -0.38); p=0.012<br>• SF12 physical (0-100): 13.2 (-2.3; 33.0) vs 10.6 (-11.2; -22.7); p=0.015<br>• SF12 mental (0-100): 5.5 (-8.5; -20.1) vs 4.3 (-9.3; -27.4); p=0.83<br>• EQ-5D (0-1): 0.22 (-0.05; -0.67) vs 0.20 (-0.06; -0.56); p=0.095		
**CERTOLIZUMAB PEGOL**			
Emery<br>2017 [33]	<u>CZP+MTX vs PBO+MTX</u><br>W 52:<br>• Remission maintained (DAS28-ESR<2.6): 28.9% vs 15.0%; p<0.001<br>• Low disease activity maintained (DAS≤3.2): 43.8% vs 28.6%; p<0.001<br>• ACR50: 61.8% vs 52.6%; p=0.023<br>• Δ HAQ-DI: -1.00 vs -0.82; p<0.001<br>• HAQ-DI≤0.5: 48.1% vs 35.7%; p=0.002<br>• Δ mTSS: 0.2 vs 1.8; p<0.001<br>• DAS28-ESR<2.6: 42.6% vs 26.8%; p<0.001<br>• ACR-EULAR remission: 32.4% vs 20.7%; p=1.8<br>• CDAI≤2.8: 38.9% vs 26.3%; p=0.001<br>• SDAI≤3.3: 38.9 vs 24.9; p<0.001<br><br>ACR response: CZP+MTX vs PBO+MTX W 2; 12; 24; 52<br>• ACR20: (51.9% vs 25.8%)*; (73.3% vs 69.5%); (70.8% vs 68.1%); (69.0% vs 61.5%)<br>• ACR50: (18.5% vs 3.3%)*; (51.0% vs 40.8%)*; (56.5% vs 50.2%); (61.8% vs 52.6%)*<br>• ACR70: (6.1% vs 0.5%)*; (33.1% vs 19.7%)* (41.1% vs 29.1%)*; (51.3% vs 39.9%)*<br>*p<0.05<br><br>Significant ↓ disease activity from w 2 to 52 CZP+MTX vs PBO+MTX w 2, 12, 24 and 52<br>• DAS28-ESR: (5.1±1.3 vs 5.9±1.3); (3.9±1.4 vs 4.4 ±1.5); (3.5±1.5 vs 4.1±1.5); (3.1±1.6 vs 3.8±1.7)<br>• CDAI: (23.7±12.9 vs 31.5±14.8); (13.3±12.0 vs 17.2±13.7); (10.8±11.3 vs 14.1±13.0); (8.7±11.6 vs 14.0±14.3)	N (%)-incidence (IC95%): PBO+MTX vs CZP+MTZ<br>Any EA (≥5%): 158 (72.8%)-195 (166.3-228-7) vs 527 (79.7%)-250.8 (229273)<br>Serious AE: 20 (9.2%)-10.7 (6.6-16.6) vs 70 (10.6%)-12.1 (9.4-15.2)	CZP+dose-optimised MTX treatment of DMARD-naïve early RA resulted in significantly more patients achieving sustained remission and sustained low disease activity, improved physical function and inhibited structural damage compared with PBO+dose-optimised MTX.

	• SDAI: (24.4±13.1 vs 33.4±15.8); (14.1±12.6 vs 18.3±14.4); (11.5±12.0 vs 15.1±13.6); (9.4±12.4 vs 14.0±14.3)<br>• SJC: (6.8±4.9 vs 9.0±6.0); (3.2±4.4 vs 5.1±5.4); (2.6±3.8 vs 3.9±4.6); (2.1±3.9 vs 3.6±4.5)<br>• ESR: (29 vs 41); (22 vs 30); (20 vs 30); (17 vs 21.5)<br>• CRP: (2.17 vs 8.35); (2.33 vs 4.69); (2.05 vs 3.74); (2.07 vs 3.96)					
Hetland 2020 [20]	W 24<br><br>Remission CDAI: % (IC 95%)<br><br>Rate differences (%- IC95%)<br>• ACR/EULAR<br>• Remission DAS28<br>• Remission SDAI<br>W 12<br>• Remission CDAI<br>• ACR/EULAR<br>• Remission DAS28<br>• Remission SDAI	CZP+MTX vs TC<br><br>46.5% (39.9-53.1)<br><br>3.9 (-5.5;10.3)<br>3.6 (-5.7;12.9)<br>2.6 (-6.6; 11.4)<br>6.4 (-3;15.7)<br><br>4.5 (-4.4;13.3)<br>7.1 (-1.3;15.6)<br>5.8 (-3.3;10.9)<br>6.9 (-2;15.7)	ABA+MTX vs TC<br><br>52.0% (45.5-58.6)<br><br>9.4 (0.1;18.7)<br>4.6 (-4.6;13.9)<br>4.5 (-4.2;13.2)<br>8.9 (-0.3;18.2)<br><br>2.6 (-6.1;11.4)<br>7.2 (-1.2;15.7)<br>2.2 (-6.9;11.3)<br>3.6 (-5.2;12.3)	TCZ+MTX vs TC<br><br>42.1% (35.3-48.8)<br><br>-0.6 (-0.1;8.9)<br>-3.8 (-13.2;5.6)<br>-0.7 (-0.8;8.4)<br>1.4 (-8.1;10.9)<br><br>4.6 (-4.4; 13.7)<br>9.2 (0.5;18)<br>14 (4.8;23.1)<br>7.5 (-1.5;16.6)	TC (n=197) vs CZP+MTX (n=202) vs ABT+MTX (n=204) vs TCL+MTX (n=184)<br><br>N.º events (nº patients; % patients)<br>• AE: 562 (170; 86.3) vs 530 (167; 82.7) vs 527 (163; 79.9)<br>• Serious: 13 (11; 5.6) vs 20 (17; 8.4) vs 10 (10; 4.9) vs 10 (9; 4.9)<br><br>Special interest AE:<br>• Infections: 93 (68; 34.5) vs 103 (74; 36.6) vs 102 (70; 34.3) vs 126 (84; 45.7)<br>• Cardiovascular: 3 (3; 1.5) vs 8 (7; 3.5) vs 10 (9; 4.4) vs 6 (6; 3.3)<br>• Diabetes: 2 (2; 1) vs - vs - vs -<br>• Herpes zoster 3 (3; 1.5) vs 1 (1; 0.5) vs – vs -<br>• Cancer: - vs 1 (1; 0.5) vas 2 (2; 1) s 3 (3; 1.6)<br>• Osteoporosis: 1 (1; 0.5) vs 3 (3; 1.5) vs – vs 1 (1; 0.5)	All four treatments achieved high remission rates. Higher CDAI remission rate was observed for abatacept versus active conventional treatment, but not for certolizumab pegol or tocilizumab versus active conventional treatment. Other remission rates were similar across treatments. Non-inferiority analysis indicated that active conventional treatment was non-inferior to certolizumab pegol and tocilizumab, but not to abatacept. The results highlight the efficacy and safety of active conventional treatment based on methotrexate combined with corticosteroids, with nominally better results for abatacept, in treatment naive early rheumatoid arthritis
Weinblatt_2017 [21]	W 104				CZP (standard dose) vs CPZ (optimization) vs CZP (stop) vs MTX	The study failed to meet its primary

	CZP (standard dose) vs CPZ (optimization) vs CZP (stop); p value (standard-stop), p value (optimization-stop)<br>• Low activity: 48.8% vs 53.2% vs 39.2%; p=0.112 and p=0.041<br>• Maintained remission 44% vs 43.4% vs 33.3%. p=0.274 and p=0.253<br>• Radiographic progression: 9.7% vs 15.9% vs 18.9%<br>• No progression (Δ SHS≤0.5): 79.2% vs 77.9% vs 70.3%)<br>• Flares according to patients: 8.3% vs 2.4% vs 12.7%<br>• HAQ-DI ≤0.5: 71.4% vs 70.6 % vs 57.0%		responders vs CPZ at any time (n (%) /incidence)<br><br>Any AE (≥5%): 53 (63.9) vs 81 (63.8) vs 48 (59.3) vs 33 (50.0) vs 145 (65.0) /163.5<br>Gastrointestinal: 5 (6.0) vs 6 (4.7) vs 8 (9.9) vs 7 (10.6) vs 13 (5.8) /6.9<br>Infections: 26 (31.3) vs 49 (38.6) vs 22 (27.2) vs 10 (15.2) vs 82 (36.8) /57.8<br>Neurologic: 6 (7.2) vs 7 (5.5) vs 1 (1.2) vs 1 (1.5) vs 15 (6.7)/14.1<br>Renal: 5 (6.0) vs 2 (1.6) vs 0 vs 1 (1.5) vs 15 (6.7)/14.1<br>Respiratory: 5 (6.0) vs 8 (6.3) vs 3 (3.7) vs 1 (1.5) vs 13 (5.8)/6.9<br>Skin: 8 (9.6) vs 9 (7.1) vs 3(3.7) vs 2 (3.0) vs 17 (7.6)/8.6<br>Cancer: 0 vs 3 (2.4) vs 2 (2.5) vs 0 vs 4 (1.8)/1.7<br><br>Serious AE: 4 (4.8) vs 9 (7.1) vs 6 (7.4) vs 4 (6.1) vs 16 (7.2)/6.9	end point. However, there were no clinically meaningful differences between the standard and reduced frequency doses of CZP plus MTX; both controlled RA more effectively than stopping CZP.
**ETANERCEPT**				
Nam_2014<br>[22]	W 52: <u>MTX+PBO vs MTX+ETN</u><br>(value; OR (95% CI); p value)<br>• No swollen or painful joints: 28.1% vs 32.5%; 1.32 (0.56-3.09); NS<br>• Remission ACR: 22.5% vs 26.7%; 1.24 (0.49-3.12); p>0.05<br>• Remission (SDAI≤3.3): 37.0% vs 47.5%; 1.57 (0.68-3.61); p>0.05<br>• DAS44-CRP: -1.32 vs -1.45; -0.11 (0.47-0.26); NS	W 72: <u>MTX+PBO vs MTX+ETN</u><br>(value; OR (95% CI); p value)<br>• No swollen or painful joints: 28.1% vs 24.6%; 0.94 (0.37-2.41); p>0.05<br>• Remission ACR: 20.5% vs 20.9%; 1.04 (0.37-2.89); p>0.05<br>• Remission SDAI: 38.5% vs 39.5%; 1.08 (0.45-2.59); p>0.05<br>• DAS44-CRP: -1.33 vs -1.29; 0.05 (-0.34-0.44); p>0.05	MTX+PBO vs MTX+ETN<br><br>Any AE n (%): 55 (100%) vs 53 (96.4%)<br>Total number AE: 338 vs 358<br>Nº AE/100 patient-year: 417.3 vs 451.6<br><br>Severity of AE<br>• Mild: 231/338 (68.3%) vs 236/358 (65.9%)<br>• Moderate: 104/338 (30.8%) vs 107/358 (29.9%)	Patients with early inflammatory arthritis, almost a third had no tender, swollen joints after 1 year. MTX+ETN was not superior to MTX monotherapy in achieving this outcome. Clinical responses, however, including DAS28-CRP<2.6, were achieved earlier with MTX+ETN combination therapy.

	• HAQ-DI: -0.31 vs -0.40; -0.09 (-0.29-0.11); NS<br>• SF-36 mental: 2.99 vs 0.97; 0.29 (-4.05-4.64); NS<br>• SF-36 physical: 6.39 vs 8.10; 2.17 (-4.17-4.60); p>0.05<br>• EQ5D-3L: 0.113 vs 0.128; 0.052 (-0.063-1.166); p>0.05<br>• Erosions: 0.37 vs 0.23; -0.13 (-0.44-0.18); p>0.05<br>• Joint space narrowing: 0.54 vs 0.68; -0.25 (-0.24-0.74); p>0.05<br>• mTSS: 0.91 vs 0.90; 0.12 (-0.47-0.72); p>0.05	• HAQ-DI: -0.37 vs -0.34; -0.04 (-0.17-0.26); NS<br>• SF-36 MCS: 2.94 vs 2.48; 1.42 (-3.10-5.95); p>0.05<br>• SF-36 PCS: 7.06 vs 6.50; -0.98 (-4.77-2.80); p>0.05<br>• EQ5D-3L: 0.151 vs 0.102; -0.029 (-0.130-0.073); p>0.05<br>• Erosions: 0.60 vs 0.50; -0.029 (-0.130-0.073); p>0.05<br>• Joint space narrowing: 0.99 vs 0.87; -0.03 (-0.52- 0.46); p>0.05	• Serious 3/338 (0.9%) vs 15/338 (4.2%)<br><br>Total number serious AE: 3 vs 13	
**INFLIXIMAB**				
Bejarano_2010 [23]	8 yr: <u>INF+MTX vs MTX+PBO</u><br><br>• DAS-28: [2.7 (2.2-3.7)] vs [4.3 (3.3-5.3)]; p=0.02<br>• CRP: [5 (5-11)] vs [6 (5-12)]; p=0.1<br>• HAQ: [1.0 (0.1-1.8)] vs [1.5 (1.2-2.1)]; p=0.12<br>• RAQoL: [3 (1-18)] vs [8 (5-27)]; p=0.1<br>• Remission (DAS28≤2.6): 4 vs 0<br>• Remission without treatment: 1 vs 0		-	A remission induction regime with an INF–MTX combination for 1 year in early RA can improve long-term clinical outcomes. Larger studies will be required to confirm the implications of these findings.
Durez_2007 [24]	Δ basal-18 w and Δ basal-52 w: Median (ICR)<br><br>- Synovitis: <u>MTX vs MP-IV vs IFX</u><br>• Δ basal-18 w: [-1(2)] vs [-3(10)] vs [-7(8)]; IFX vs MTX: p<0.05<br>• Δ basal-52 w: [-4.5(9)] vs [-8(13)] vs [-10.5(11)]; IFX vs MTX: p<0.05<br>- Edema: MTX vs MP-IV vs IFX<br>• Δ basal-18 w: [-2(7)] vs [-2(6)] vs [-5.5(15)]; IFX vs MTX: p<0.05<br>• Δ basal-52 w: [-2(6)] vs [-2(5)] vs [-9(20)]; IFX vs MTX: p<0.05<br>- Erosions: MTX vs MP-IV vs IFX<br>• Δ basal-18 w: [1(2)] vs [3(5)] vs [1(2)]<br>• Δ basal-52 w: [1(6)] vs [6(10)] vs [1(3)]; MP-IV vs MTX: p<0.05		N=1 serious AE in MTX+IFX at w 30	The combination of MTX and infliximab is superior to MTX alone for reducing MRI-detected signs of synovitis and bone edema in patients with early RA. Progression of MRI-detected erosion was greater in patients treated with MTX plus IV MP compared with that in patients who received MTX plus infliximab.

	Δ DAS-28-CRP MTX vs MTX+MPIV vs MTX+IFX (w 52)<br>↓4.85±0.96 to 3.26±1.32 (p=0.005) vs ↓5.39±1.22 to 2.77±1.09 (p<0.0001) vs ↓5.57±1.03 to 2.79±0.77 (p<0.0001)<br><br>ACR20/50/70 response:<br>W 22: MP-IV and IFX vs MTX: p<0.005<br>W 52: no differences<br><br>Remission EULAR (w 52): MP-IV (70%) vs MTX (40%); p<0.05		
Goekoop-Ruiterman_2005 [25]	<u>Monotherapy sequential vs combination sequential vs combination with prednisone vs combination with IFX</u><br><br>- D-HAQ<br>• Basal: (1.4±0.7) vs (1.4± 0.6) vs (1.4 ±0.7) vs (1.4± 0.7); p>0.05<br>• M 3: (1.0± 0.7) vs (1.0± 0.6) vs (0.6± 0.6) vs (0.6±0.6); p<0.001 group 1 and 2 vs 3 and 4<br>• M 6: (0.9± 0.7) vs (0.9± 0.7) vs (0.5± 0.5) vs (0.5± 0.5); p<0.001 group 1 and 2 vs 3 and 4<br>• M 9: (0.8± 0.7) vs (0.8± 0.7) vs (0.6± 0.6) vs (0.5± 0.6); p=0.001 group 1 and 2 vs 3 and 4<br>• M 12: (0.7±0.7) vs (0.7± 0.6) vs (0.5± 0.5) vs (0.5±0.5); p=0.009 group 1 and 2 vs 3 and 4<br><br>- Radiographic progression:<br>• SHS total (0–448): (7.1±15.4) vs (4.3±6.5) vs (2.0±3.6) vs (1.3±4.0); p<0. group 1 and 2 vs 3 and 4<br>• Erosions (0–280): (3.5± 8.2) vs (2.6±4.7) vs (0.9±1.9) vs (0.7± 2.1); p<0.001 group 1 and 2 vs 3 and 4<br>• Joint space narrowing (0–168): (3.6±8.4) vs (1.6±2.9) vs (1.0±2.4) vs (0.6±2.6); p<0.001 group 1 and 2 vs 3 and 4	<u>Monotherapy sequential vs combination sequential vs combination with prednisone vs combination with IFX</u><br><br>AE≥1: 54 (43%) vs 57(43%) vs 49 (37%) vs 50 (39%); p=0.367<br>• Gastrointestinal: 20 (16%) vs 18 (15%) vs 11 (8%) vs 14 (11%)<br>• Skin: 12 (10%) vs 15 (12%) vs 12 (9%) vs 8 (6%)<br>• Respiratory infections: 5(4%) vs 8(7%) vs 10 (8%) vs 10 (8%)<br>• Cardiovascular: 3 (2%) vs 2 (2%) vs 8 (6%) vs 2 (2%)<br>• TBC (latent): 9 pacientes group 4<br><br>Serious AE: 8 vs 9 vs 17 vs 6; p=0.438	In patients with early RA, initial combination therapy including either prednisone or infliximab resulted in earlier functional improvement and less radiographic damage after 1 year than did sequential monotherapy or step-up combination therapy.
Goekoop-Ruiterman_2007 [26]	Best Study: 2nd year<br><u>Monotherapy sequential vs combination sequential vs combination with prednisone vs combination with IFX</u>	Best Study: 2nd year<br><u>Monotherapy sequential vs combination sequential vs combination</u>	Currently available antirheumatic drugs can be highly effective in patients with early rheumatoid arthritis in a setting of

	- HAQ (Δ from baseline):<br>• M 3: (0.4±0.6) vs (0.3±0.6) vs (0.8±0.7) vs (0.7±0.6); p<0.001<br>• M 6: (0.5±0.7) vs (0.5±0.7) vs (0.9±0.7) vs (0.8±0.6); p<0.001<br>• M 9: (0.6±0.7) vs (0.6±0.7) vs (0.8±0.7) vs (0.8±0.6); p= 0.010<br>• M 12: (0.7±0.7) vs (0.7±0.7) vs (0.9±0.7) vs (0.9±0.7); p= 0.031‡<br>• M 15: (0.7±0.7) vs (0.8±0.7) vs (0.7±0.8) vs (0.9±0.7); p=0.299<br>• M 18: (0.7±0.7) vs (0.8±0.7) vs (0.8±0.8) vs (0.9±0.7); p=0.255<br>• M 21: (0.7±0.7) vs (0.8±0.7) vs (0.8±0.7) vs (0.9±0.7); p=0.220<br>• M 24: (0.7±0.7) vs (0.8±0.7) vs (0.9±0.7) vs (0.9±0.7); p=0.257<br><br>- Radiographic progression (Δ from baseline):<br>• SHS total: (9.0±17.9) vs (5.2±8.1) vs (2.6±4.5) vs (2.5±4.6); p=0.005<br>• Erosions: (7±9.0) vs (3.1±5.0) vs (1.1±2.2) vs (1.3±2.7); p<0.001<br>• Joint space narrowing: (4.3±9.8) vs (2.1±3.8) vs (1.5±3.2) vs (1.2±2.9); p= 0.072<br><br>- Disease activity (≤2.4 m 6 and 24): 22% vs 21% vs 28% vs 40%	with prednisone vs combination with IFX<br><br>Any AE<br>• Gastrointestinal: 14 (12%) vs 11 (9%) vs 12 (9%) vs 15 (12%)<br>• Skin: 12 (10%) vs 10 (8%) vs 15 (11%) vs 7 (6%)<br>• Infections: 10 (8%) vs 10 (8%) vs 10 (8%) vs 13 (10%)<br>• Cardiovascular: 5 (4%) vs 5 (4%) vs 9 (7%) vs 8 (6%)<br><br>Serious AE<br>• 1st year: 8 vs 9 vs 17 vs 6 patients<br>• 2nd year (n=56): 16 (13 patients] vs 10 (10 patients) vs 17 (11 patients) vs 13 (8 patients)]	tight disease control. Initial combination therapies seem to provide earlier clinical improvement and less progression of joint damage, but all treatment strategies eventually showed similar clinical improvements. In addition, combination therapy can be withdrawn successfully and less treatment adjustments are needed than with initial monotherapies.
Goekoop-Ruiterman_2008 [27]	Best Study: 2nd year<br>Monotherapy sequential vs combination sequential vs combination with prednisone vs combination with IFX<br>- DAS44≤2.4: 63 (53%) vs 72 (64%) vs 87 (71%) vs 89 (74%); (p=0.004 group 1 vs 3; p=0.01 group 1 vs 4; rest p>0.05)<br><br>- D-HAQ<br>• Basal: (1.4±0.7) vs (1.4±0.6) vs (1.4±0.7) vs (1.4±0.7); p=NS<br>• M 2: (1.0±0.7) vs (1.0±0.6) vs (0.6±0.6) vs (0.6±0.6); p<0.001<br>• M 6: (0.9±0.7) vs (0.9±0.7) vs (0.5±0.5) vs (0.5±0.5); p<0.001<br>• M 9: (0.8±0.7) vs (0.8±0.7) vs (0.6±0.6) vs (0.5±0.6); p=0.001<br>• M 12: (0.7±0.7) vs (0.7±0.6) vs (0.5±0.5) vs (0.5±0.5); p=0.009<br><br>- Radiographic progression: SHS	Best Study: 2nd year<br>Monotherapy sequential vs combination sequential vs combination with prednisone vs combination with IFX<br><br>• EA ≥1: 43% vs 47% vs 37% vs 39%; p=0.367<br><br>EA more prevalent:<br>• Gastrointestinal: 16% vs 15% vs 8% vs 11%<br>• Skin: 10% vs 12% vs 9% vs 6%<br>• Infections: 4% vs 7% vs 8% vs 8%	In patients with early RA, initial combination therapy including either prednisone or infliximab resulted in earlier functional improvement and less radiographic damage after 1 year than did sequential monotherapy or step-up combination therapy.

	• No progression (total): 67% vs73% vs 87% vs 93%; p<0.001(group 1 vs 3-4); p=0.010 (2 vs 3); p<0.001 (2 vs 4)<br>• Erosions (0–280): (3.5± 8.2) vs (2.6±4.7) vs (0.9±1.9) vs (0.7± 2.1); p<0.001<br>• Joint space narrowing (0–168): (3.6±8.4) vs (1.6±2.9) vs (1.0±2.4) vs (0.6±2.6); p<0.001	• Cardiovascular: 2% vs 2% vs 6% vs 2%<br>• No TBC<br><br>Serious AE: 8. 9. 17 y 6 (p=0.438)	
Markusse_2016 [28]	Best Study: 10 year<br>Monotherapy sequential vs combination sequential vs combination with prednisone vs combination with IFX<br><br>- DAS44≤2.4: 84% vs 77% vs 83% vs 84%; p=0.72<br>- Remission: 51% vs 49% vs 53% vs 53%. p=0.94<br>- Remission without treatment at least 1 visit: 27% vs 24% vs 22% vs 29%<br>• Radiographic progression: Δ SHS [2.0 (0-11.0)] vs [2.5 (0-13.5)] vs [3.0 (0.3-11.3)] vs [1.5 (0.0-6.0)]<br><br>-Year 10: Patients with initial treatment n (%): 21 (17%) vs 13 (11%) vs 33 (25%) vs 52 (41%)	Best Study: 2nd year<br>Monotherapy sequential vs combination sequential vs combination with prednisone vs combination with IFX<br><br>AE: 3109 in 453 patients (89%) during 10 yr:<br>• Incidence: 74/100 patient-year<br>• No differences by group (p=0.159)<br>• Most frequent infections withoud differences among groups (p=0.158)<br>• Group 3: > frequency of cardiovascular AE<br><br>Serious AE: 486 in 240 patients (47%)<br>• Incidence: 12/100 patient-year<br>• No differences by group (p=0.47)	In patients with early RA, initial (temporary) combination therapy results in faster clinical improvement and targeted treatment determines long-term outcomes. Drug-free remission, with prevention of functional deterioration and clinically relevant radiographic damage, and normalized survival are realistic outcomes
Nam_2014 [29]	MP-IV vs IFX<br>- Radiographic progression (w 50)<br>• Δ mTSS: 2.81±6.88 vs 1.20±2.27; p=0.132<br>• Δ erosion: 11.28±3.26 vs 0.49±1.21; p=0.224<br>• Δ Joint space narrowing: 1.53±3.95 vs 0.71±1.31; p=0.141<br><br>- Radiographic progression (w 78)<br>• Δ mTSS: 3.19±7.75 vs 1.69±3.28; p=0.253<br>• Δ erosion: 1.32±3.46 vs 0.75±2.03; p=0.564<br>• Δ Joint space narrowing: 1.87±4.58 vs 0.94±1.69; p=0.178	MP-IV vs IFX:<br><br>Patients with any AE: 54 (97.4%) vs 54 (98.2%)<br>Total number of any AE: 372 vs 369<br><br>AE severity<br>• Mild: 230/372 (61.8%) vs 226/369 (73.2%)<br>• Moderate: 133/372 (35.8%) vs 122/369 (33.1%)	In DMARD-naive early RA patients, initial therapy with MTX+high-dose intravenous steroid resulted in good disease control with little structural damage. MTX +IFX was not statistically superior to MTX+intravenous steroid when combined with a treat-to-target approach

	Remission: %; OR (95% CI); p value<br>- W 6<br>• Remission (DAS44): 7.1% vs 18.3%; 5.02 (1.30-19.33); p=0.019<br>• Low disease activity (DAS44): 30.4% vs 46.0%; 3.75 (1.46-9.62); p=0.006<br>• Remission (DAS28): 7.5% vs 22.3%; 6.16 (1.61-23.54); p=0.008<br>• Low disease activity (DAS28): 23.9% vs 33.5%; 2.63 (1.00-6.93); p=0.051<br>• Remission (SDAI): 3.6% vs 4.4%; 1.54 (0.21-11.48); p=0.674<br><br>- W 14<br>• Remission (DAS44): 31.0% vs 34.8%; 1.89 (0.78-4.62); p=0.161<br>• Low disease activity (DAS44): 61.4% vs 66.8%; 3.44 (1.21-9.76); p=0.020<br>• Remission (DAS28): 40.0% vs 42.3%; 1.68 (0.70-4.05); p=0.250<br>• Low disease activity (DAS28): 54.1% vs 55.4%; 1.61(0.67-3.83); p=0.285<br>• Remission (SDAI): 16.7% vs 24.5%; 2.04 (0.70-5.92); p=0.191<br><br>- W 26<br>• Remission (DAS44): 40.0% vs 31.6%; 0.82 (0.36-1.88); p=0.644<br>• Low disease activity (DAS44): 68.4% vs 65.9%; 1.48 (0.57-3.84); p=422<br>• Remission (DAS28): 50.8% vs 40.6%; 0.76 (0.34-1.70); p=0.510<br>• Low disease activity (DAS28): 66.6% vs 64.6%; 1.32 (0.54-3.27); p=0.545<br>• Remission (SDAI): 24.1% vs 29.6%; 1.55 (0.63-3.79); p=0.340<br>• Remission (ACR-EULAR): 19.3% vs 14.5%; 0.75 (0.27-2.08); p=0.587<br>• ACR20: 75.2% vs 71.0%; 0.81 (0.35-1.89); p=0.626<br>• ACR50: 55.1% vs 54.0%; 0.95 (0.45-2.03); p=0.898<br>• ACR75: 31.8% vs 32.7%; 1.04(0.46-2.33); p=0.927<br>• Δ HAQ-DI (mean difference): -0.61±0.47 vs -0.70±0.56; -0.05 (-0.23. 0.13); p=0.568<br><br>- W 50<br>• Remission (DAS44): 36.1% vs 48.5%; 2.13 (0.91-5.00); p=0.082<br>• Low disease activity (DAS44): 70.6% vs 70.4%; 1.26 (0.51-3.12); p=0.617<br>• Remission (DAS28): 49.6% vs 55.7%; 1.71(0.73-3.99); p=0.218<br>• Low disease activity (DAS28): 63.4% vs 68.7%; 1.68 (0.68-4.19); p=0.264<br>• Remission (SDAI): 27.2% vs 45.3%; 2.98 (1.18-7.56); p=0.021<br>• Remission (ACR-EULAR): 19.4% vs 16.5%; 0.84(0.31-2.25); p=0.729	• Serious 9/372 (2.4%) vs 21/369 (5.7%)<br><br>Number of serious AE: 9 vs 20<br>• TBC n=1 with IFX<br>• Herpes zoster n=1 with MP-IV	

	- W 78<br>• Remission (DAS44): 50.0% vs 47.7%; 1.12 (0.47-2.68); p=0.795<br>• ACR50: 80.4% vs 72.7%; 0.92 (0.29-2.93); p=0.888<br>• Low disease activity (DAS44): 80.4% vs 72.7%; 0.92 (0.29-2.93); p=0.888<br>• Remission (DAS28): 65.3% vs 54.3%; 0.74 (0.31-1.76); p=0.4888<br>• Low disease activity (DAS28): 76.4% vs 63.3%; 0.60 (0.23-1.53); p=0.282<br>• Remission (SDAI): 49.4% vs 37.6%; 0.73 (0.30-1.76); p=0.480<br>• Remission (ACR-EULAR): 15.9% vs 15.7%; 1.04 (0.35-3.08); p=0.950<br>• ACR20: 71.1% vs 70.7%; 0.98 (0.39-2.46); p=0.973<br>• ACR50: 63.4% vs 64.3%; 1.03 (0.43-2.48); p=0.953<br>• ACR75: 50.1% vs 46.2%; 0.84(0.37-1.88); p=0.669<br>• Δ HAQ-DI (mean difference): -0.79±0.54 vs -0.85±0.60; - 0.03(-0.26-0.21); p=0.826<br>• Δ RAQoL (mean difference): -7.96±6.32 vs -9.71±7.08; - 0.62 (-0.34-2.09); p=0.650		
Quinn_2005 [30]	IFX+MTX vs PBO+MTX<br><br>- Remission (ACR): 7 patients vs 2 patients (w 104); Time (median) in remission: 26 w vs 0 w (p<0.05)<br><br>- Activity:<br>• CRP: ↓ favoring IFX up to w 54 (p<0.05)<br>• ↓ DAS28 (p<0.005) in IFX vs PBO in w 14, 54 and 104<br><br>- MRI (median)<br>• Synovitis: (↓5.5 to 3.4) vs (↓6.2 to 5.9); p<0.05 (w 14) and values 3.8 vs 6.6; p<0.05 (w 54)<br>• Edema: better with IFX (p<0.05) in w 4, 14 and 54<br>• Erosions: ↓ significant with IFX+MTX in w 24 (p=0.012)<br><br>- SHS): no differences (10 vs 12) at m 24<br><br>- ACR response<br>W 14<br>• ACR20: 60% vs 20% p<0.05	-	Remission induction with infliximab plus MTX provided a significant reduction in MRI evidence of synovitis and erosions at 1 year. At 2 years, functional and quality of life benefits were sustained, despite withdrawal of infliximab therapy. These data may have significant implications for the optimal use of expensive biologic therapies

	• ACR50: 60% vs 0%; p<0.05<br>• ACR70: 60% vs 0%; p<0.05<br>W 54<br>• ACR20: 80% vs 60%<br>• ACR50: 80% vs 40%; p<0.05<br>• ACR70: 70% vs 30%; p<0.05<br>W 104<br>• ACR20: 70% vs 50%; NS<br>• ACR50: 70% vs 50%; NS<br>• ACR70: 50% vs 50%; NS<br><br>- Function and quality of life<br>• HAQ: Improvement (p<0.005) favoring IFX in w 14, 54 and 104<br>• RAQoL: Improvement (p<0.005) favoring IFX in w 14, 54 and 104		
**TOCILIZUMAB**			
TCZ<br>Bijlsma_2016<br>[31]	TCZ+MTX vs TCZ vs MTX<br><br>Remission maintained (DAS28)<br>Initial treatment: 86% vs 83% vs 44%; TCZ+MTX vs MTX p<0.0001; TCZ vs MTX p<0.0001; TCZ+MTX vs TCZ p=0.062<br>All the study 86% vs 88% vs 77%; TCZ+MTX vs MTX p=0.06; TCZ vs MTX p=0.035; TCZ+MTX vs TCZ p=0.059<br><br>EULAR response<br>W 24:<br>• Good 89% vs 87% vs 49%: TCZ+MTX vs MTX: p<0.0001; TCZ vs MTX: p<0.0001. TCZ+MTX vs TCZ: p=0.43<br>• Moderate: 5% vs 11% vs 32%: TCZ+MTX vs MTX: p<0.0001; TCZ vs MTX: p<0.0001. TCZ+MTX vs TCZ: p=0.43<br><br>W 52:<br>• Good 75% vs 88% vs 72%: TCZ+MTX vs MTX: p=0.26; TCZ vs MTX: p=0.0074; TCZ+MTX vs TCZ: p=0.06	TCZ+MTX vs TCZ vs MTX<br><br>• Any EA: 105 (99.1%) vs 99 (96.1%) vs 106 (98.1%). p=0.32<br>• Serious AE: 17 (16.0%) vs 19 (18.4%) vs 13 (12.0%). p=0.44<br>• Serious infections: 4 (3.8%) vs 6 (5.8%) vs 5 (4.6%). p=0.76	For patients with newly diagnosed rheumatoid arthritis, strategies aimed at sustained remission by immediate initiation of tocilizumab with or without methotrexate are more effective, and with a similar safety profile, compared with initiation of methotrexate in line with current standards.

	• Moderate 6% vs 4% vs 7%: TCZ+MTX vs MTX: p=0.26; TCZ vs MTX: p=0.0074; TCZ+MTX vs TCZ: p=0.06<br><br>W 104:<br>• Good 66% vs 76% vs 68%: TCZ+MTX vs MTX: p=0.87; TCZ vs MTX: p=0.13; TCZ+MTX vs TCZ: p=0.10<br>• Moderate 8% vs 8% vs 8%: TCZ+MTX vs MTX: p=0.87; TCZ vs MTX: p=0.13; TCZ+MTX vs TCZ: p=0.10<br><br>ACR20/50/70/90 response<br>W 24<br>• ACR20 TCZ+MTX vs MTX p=0.0099; TCZ vs MTX p=0.0343<br>• ACR50 TCZ+MTX vs MTX p<0.0001; TCZ vs MTX p=0.0009<br>• ACR70 TCZ+MTX vs MTX p<0.0001; TCZ vs MTX p=0.0003<br>• ACR90 TCZ+MTX vs MTX p=0.0027<br><br>W 52:<br>• ACR90 TCZ+MTX vs MTX p=0.0045; TCZ vs MTX p=0.0026<br><br>W 104: no differences<br><br>HAQ<br>• W 24: 0.50±0.55 vs 0.63±0.66 vs 0.65±0.54; p=0.0275<br>• W 52: 0.46±0.50 vs 0.48±0.55 vs 0.55±0.51; p=0.14<br>• W 104: 0.48±0.55 vs 0.61±0.61 vs 0.62±0.50; p=0.06<br><br>Δ mTSS media (DE)/median (ICR)<br>• W 52: 0.50±1.495 vs 0.79±3.242 vs 0.96±2.870; TCZ+MTX vs MTX p<0.0164; TCZ vs MTX p=0.06; TCZ+MTX vs TCZ p=0.49<br>• W 104: 1.18±3.919 vs 1.45±4.272 vs 1.53±2.421; TCZ+MTX vs MTX p=0.0207; TCZ vs MTX p=0.0381; TCZ+MTX vs TCZ p=0.53<br><br>Maintained remission without treatment during 3 m:<br>• 35% vs 27% vs 11%; TCZ+MTX vs MTX p<0.0001; TCZ vs MTX p=0.0037; TCZ+MTX vs TCZ p=0.28		

	Maintained remission without treatment (w):<br>• [44 (1-56)] vs [32 (20-48)] vs [28 (20-44)]: TCZ+MTX vs MTX: p=0.10; TCZ vs MTX: p=0.50; TCZ+MTX vs TCZ p=0.16		
Verhoeven_2020 [32]	DAS28 mean difference C95% CI)<br>During 5 yr<br>• TCZ+MTX vs MTZ -0.11 (-0.32 to 0.10)<br>• TCZ vs MTZ -0.12 (-0.32 to 0.09)<br>• TCZ+MTX vs TCZ -0.00 (-0.20 to 0.21)<br>Year 1<br>• TCZ+MTX vs MTZ -0.75 (-0.89 to -0.61)<br>• TCZ vs MTZ -0.65 (-0.79 to -0.51)<br>• TCZ+MTX vs TCZ -0.10 (-0.23 to 0.04)<br>Year 2<br>• TCZ+MTX vs MTX -063 (-0.77 to -0.49)<br>• TCZ vs MTZ -0.55 (-0.69 to -0.40)<br>• TCZ+MTX vs TCZ -0.08 (0.23 to 0.06)<br>Year 3<br>• TCZ+MTX vs MTZ: -0.07 (-0.19 to 0.33)<br>• TCZ vs MTZ: -0.09 (-0.17 to -0.34)<br>• TCZ+MTX vs TCZ: -0.02 (-0.27 to 0.24)<br>Year 4<br>• TCZ+MTX vs MTX -0.09 (-0.17 to 0.35)<br>• TCZ vs MTX -0.10 (-0.16 to 0.36)<br>• TCZ+MTX vs TCZ -0.01 (-0.27 to 0.24)<br>Year 5<br>• TCZ+MTX vs MTX -0.11 (-0.16 to 0.37)<br>• TCZ vs MTX -0.12 (-0.14 to 0.38)<br>• TCZ+MTX vs TCZ -0.01 (-0.27 to 0.25)<br><br>Maintained remission during 5 yr (n=226) TCZ+MTX vs TCZ vs MTX<br>• At least 1 n (%): 75 (100%) vs 77 (98%) vs 72 (100%); p=0.15<br>• Accumulated duration w: [216 (152-251)] vs [190 (135-240)] vs [172 (129-202]); p<0.01<br>• Without treatment at least 1 n (%): 26 (35%) vs 19 (26%) vs 14 (19%). p=0.10	TCZ+MTX vs TCZ vs MTX<br><br>No significant differences<br><br>Serious AE: 23 (28%) vs 23 (29%) vs 15 (21%)	Although in the short-term the strategies initiating TCZ yielded the most clinical benefit, in the longer-term differences in important clinical outcomes between the strategies disappeared, probably due to continuation of the treat-to-target principle.

	• Accumulated duration without treatment (w) [119 (76-157)] vs [107 (53-157)] vs [83 (37-146)]; p<0.27<br><br>Δ SvdH during 5 yr: TCZ+MTX vs TCZ vs MTX<br>• Total [0 (0-1)] vs [0 (0-1)] vs [0 (0-2)]; TCZ+MTX vs MTX p=0.41; TCZ vs MTX p=0.05; TCZ+MTX vs TCZ p=0.67<br>• Erosions [0 (0-0)] vs [0 (0-1)] vs [0 (0-1)]; TCZ+MTX vs MTX: p=0.62; TCZ vs MTX: p=0.80; TCZ+MTX vs TCZ; p=0.62<br>• Joint space narrowing [0 (0-0)] vs [0 (0-0)] vs[0 (0-1); TCZ+MTX vs MTX p=0.03; TCZ vs MTX p=0.11; TCZ+MTX vs TCZ p=0.31		

Abbreviations: ABT=abatacept; ACR=American College Rheumatology; ADA=adalimumab; CDAI=Clinical Disease Activity Index; HAQ=health assessment questionnaire; D-HAQ=Dutch version of HAQ; mTSS= van der Heijde modified total Sharpe score; MP=metilprednisolone; PBO=placebo; RAQoL=Rheumatoid Arthritis Quality of Life Scale; SDAI=Simplified Disease Activity Index; SHS= Sharp-van der Heijde index; SFZ=sulphasalazine; TCZ=Tocilizumab; TJC=tender joint count; SJC=tender joint count; m=month; yr=year; d=day; AE=adverse event; sc=subcutaneous; iv=intravenous; ESR=erythrocyte sedimentation rate; VAS visual analogic scale; CRP=C reactive protein; MRI= magnetic resonance imaging; CI=confidence interval.

References

1. Breedveld, F.C.; Weisman, M.H.; Kavanaugh, A.F.; Cohen, S.B.; Pavelka, K.; van Vollenhoven, R.; Sharp, J.; Perez, J.L.; Spencer-Green, G.T. The PREMIER study: A multicenter, randomized, double-blind clinical trial of combination therapy with adalimumab plus methotrexate versus methotrexate alone or adalimumab alone in patients with early, aggressive rheumatoid arthritis who had not had previous methotrexate treatment. *Arthritis Rheum* **2006**, *54*, 26-37, doi:10.1002/art.21519.
2. Kavanaugh, A.; Fleischmann, R.M.; Emery, P.; Kupper, H.; Redden, L.; Guerette, B.; Santra, S.; Smolen, J.S. Clinical, functional and radiographic consequences of achieving stable low disease activity and remission with adalimumab plus methotrexate or methotrexate alone in early rheumatoid arthritis: 26-week results from the randomised, controlled OPTIMA study. *Ann Rheum Dis* **2013**, *72*, 64-71, doi:10.1136/annrheumdis-2011-201247.
3. Bathon, J.M.; Martin, R.W.; Fleischmann, R.M.; Tesser, J.R.; Schiff, M.H.; Keystone, E.C.; Genovese, M.C.; Wasko, M.C.; Moreland, L.W.; Weaver, A.L.; et al. A comparison of etanercept and methotrexate in patients with early rheumatoid arthritis. *N Engl J Med* **2000**, *343*, 1586-1593, doi:10.1056/nejm200011303432201.
4. Emery, P.; Breedveld, F.C.; Hall, S.; Durez, P.; Chang, D.J.; Robertson, D.; Singh, A.; Pedersen, R.D.; Koenig, A.S.; Freundlich, B. Comparison of methotrexate monotherapy with a combination of methotrexate and etanercept in active, early, moderate to severe rheumatoid arthritis (COMET): a randomised, double-blind, parallel treatment trial. *Lancet* **2008**, *372*, 375-382, doi:10.1016/s0140-6736(08)61000-4.
5. Emery, P.; Breedveld, F.; van der Heijde, D.; Ferraccioli, G.; Dougados, M.; Robertson, D.; Pedersen, R.; Koenig, A.S.; Freundlich, B. Two-year clinical and radiographic results with combination etanercept-methotrexate therapy versus monotherapy in early rheumatoid arthritis: a two-year, double-blind, randomized study. *Arthritis Rheum* **2010**, *62*, 674-682, doi:10.1002/art.27268.
6. Kekow, J.; Moots, R.J.; Emery, P.; Durez, P.; Koenig, A.; Singh, A.; Pedersen, R.; Robertson, D.; Freundlich, B.; Sato, R. Patient-reported outcomes improve with etanercept plus methotrexate in active early rheumatoid arthritis and the improvement is strongly associated with remission: the COMET trial. *Ann Rheum Dis* **2010**, *69*, 222-225, doi:10.1136/ard.2008.102509.
7. Rantalaiho, V.; Kautiainen, H.; Korpela, M.; Hannonen, P.; Kaipiainen-Seppänen, O.; Möttönen, T.; Kauppi, M.; Karjalainen, A.; Laiho, K.; Laasonen, L.; et al. Targeted treatment with a combination of traditional DMARDs produces excellent clinical and radiographic long-term outcomes in early rheumatoid arthritis regardless of initial infliximab. The 5-year follow-up results of a randomised clinical trial, the NEO-RACo trial. *Ann Rheum Dis* **2014**, *73*, 1954-1961, doi:10.1136/annrheumdis-2013-203497.
8. St Clair, E.W.; van der Heijde, D.M.; Smolen, J.S.; Maini, R.N.; Bathon, J.M.; Emery, P.; Keystone, E.; Schiff, M.; Kalden, J.R.; Wang, B.; et al. Combination of infliximab and methotrexate therapy for early rheumatoid arthritis: a

randomized, controlled trial. *Arthritis Rheum* **2004**, *50*, 3432-3443, doi:10.1002/art.20568.
9. Tam, L.S.; Shang, Q.; Li, E.K.; Wang, S.; Li, R.J.; Lee, K.L.; Leung, Y.Y.; Ying, K.Y.; Yim, C.W.; Kun, E.W.; et al. Infliximab is associated with improvement in arterial stiffness in patients with early rheumatoid arthritis -- a randomized trial. *J Rheumatol* **2012**, *39*, 2267-2275, doi:10.3899/jrheum.120541.
10. Burmester, G.R.; Rigby, W.F.; van Vollenhoven, R.F.; Kay, J.; Rubbert-Roth, A.; Kelman, A.; Dimonaco, S.; Mitchell, N. Tocilizumab in early progressive rheumatoid arthritis: FUNCTION, a randomised controlled trial. *Ann Rheum Dis* **2016**, *75*, 1081-1091, doi:10.1136/annrheumdis-2015-207628.
11. Jones, G.; Sebba, A.; Gu, J.; Lowenstein, M.B.; Calvo, A.; Gomez-Reino, J.J.; Siri, D.A.; Tomsic, M.; Alecock, E.; Woodworth, T.; et al. Comparison of tocilizumab monotherapy versus methotrexate monotherapy in patients with moderate to severe rheumatoid arthritis: the AMBITION study. *Ann Rheum Dis* **2010**, *69*, 88-96, doi:10.1136/ard.2008.105197.
12. Kume, K.; Amano, K.; Yamada, S.; Hatta, K.; Ohta, H.; Kuwaba, N. Tocilizumab monotherapy reduces arterial stiffness as effectively as etanercept or adalimumab monotherapy in rheumatoid arthritis: an open-label randomized controlled trial. *J Rheumatol* **2011**, *38*, 2169-2171, doi:10.3899/jrheum.110340.
13. Simpson, E.L.; Ren, S.; Hock, E.S.; Stevens, J.W.; Binard, A.; Pers, Y.M.; Archer, R.; Paisley, S.; Stevenson, M.D.; Herpin, C.; et al. Rheumatoid arthritis treated with 6-months of first-line biologic or biosimilar therapy: an updated systematic review and network meta-analysis. *Int J Technol Assess Health Care* **2019**, *35*, 36-44, doi:10.1017/s0266462318003628.
14. Singh, J.A.; Hossain, A.; Mudano, A.S.; Tanjong Ghogomu, E.; Suarez-Almazor, M.E.; Buchbinder, R.; Maxwell, L.J.; Tugwell, P.; Wells, G.A. Biologics or tofacitinib for people with rheumatoid arthritis naive to methotrexate: a systematic review and network meta-analysis. *Cochrane Database Syst Rev* **2017**, *5*, Cd012657, doi:10.1002/14651858.Cd012657.
15. Stevenson, M.; Archer, R.; Tosh, J.; Simpson, E.; Everson-Hock, E.; Stevens, J.; Hernandez-Alava, M.; Paisley, S.; Dickinson, K.; Scott, D.; et al. Adalimumab, etanercept, infliximab, certolizumab pegol, golimumab, tocilizumab and abatacept for the treatment of rheumatoid arthritis not previously treated with disease-modifying antirheumatic drugs and after the failure of conventional disease-modifying antirheumatic drugs only: systematic review and economic evaluation. *Health Technol Assess* **2016**, *20*, 1-610, doi:10.3310/hta20350.
16. Detert, J.; Bastian, H.; Listing, J.; Weiß, A.; Wassenberg, S.; Liebhaber, A.; Rockwitz, K.; Alten, R.; Krüger, K.; Rau, R.; et al. Induction therapy with adalimumab plus methotrexate for 24 weeks followed by methotrexate monotherapy up to week 48 versus methotrexate therapy alone for DMARD-naive patients with early rheumatoid arthritis: HIT HARD, an investigator-initiated study. *Ann Rheum Dis* **2013**, *72*, 844-850, doi:10.1136/annrheumdis-2012-201612.
17. Soubrier, M.; Puéchal, X.; Sibilia, J.; Mariette, X.; Meyer, O.; Combe, B.; Flipo, R.M.; Mulleman, D.; Berenbaum, F.; Zarnitsky, C.; et al. Evaluation of two strategies (initial methotrexate monotherapy vs its combination with adalimumab) in management of early active rheumatoid arthritis: data from the

GUEPARD trial. *Rheumatology (Oxford)* **2009**, *48*, 1429-1434, doi:10.1093/rheumatology/kep261.
18. Hørslev-Petersen, K.; Hetland, M.L.; Junker, P.; Pødenphant, J.; Ellingsen, T.; Ahlquist, P.; Lindegaard, H.; Linauskas, A.; Schlemmer, A.; Dam, M.Y.; et al. Adalimumab added to a treat-to-target strategy with methotrexate and intra-articular triamcinolone in early rheumatoid arthritis increased remission rates, function and quality of life. The OPERA Study: an investigator-initiated, randomised, double-blind, parallel-group, placebo-controlled trial. *Ann Rheum Dis* **2014**, *73*, 654-661, doi:10.1136/annrheumdis-2012-202735.
19. Atsumi, T.; Tanaka, Y.; Yamamoto, K.; Takeuchi, T.; Yamanaka, H.; Ishiguro, N.; Eguchi, K.; Watanabe, A.; Origasa, H.; Yasuda, S.; et al. Clinical benefit of 1-year certolizumab pegol (CZP) add-on therapy to methotrexate treatment in patients with early rheumatoid arthritis was observed following CZP discontinuation: 2-year results of the C-OPERA study, a phase III randomised trial. *Ann Rheum Dis* **2017**, *76*, 1348-1356, doi:10.1136/annrheumdis-2016-210246.
20. Hetland, M.L.; Haavardsholm, E.A.; Rudin, A.; Nordström, D.; Nurmohamed, M.; Gudbjornsson, B.; Lampa, J.; Hørslev-Petersen, K.; Uhlig, T.; Grondal, G.; et al. Active conventional treatment and three different biological treatments in early rheumatoid arthritis: phase IV investigator initiated, randomised, observer blinded clinical trial. *Bmj* **2020**, *371*, m4328, doi:10.1136/bmj.m4328.
21. Weinblatt, M.E.; Bingham, C.O., 3rd; Burmester, G.R.; Bykerk, V.P.; Furst, D.E.; Mariette, X.; van der Heijde, D.; van Vollenhoven, R.; VanLunen, B.; Ecoffet, C.; et al. A Phase III Study Evaluating Continuation, Tapering, and Withdrawal of Certolizumab Pegol After One Year of Therapy in Patients With Early Rheumatoid Arthritis. *Arthritis Rheumatol* **2017**, *69*, 1937-1948, doi:10.1002/art.40196.
22. Nam, J.L.; Villeneuve, E.; Hensor, E.M.; Wakefield, R.J.; Conaghan, P.G.; Green, M.J.; Gough, A.; Quinn, M.; Reece, R.; Cox, S.R.; et al. A randomised controlled trial of etanercept and methotrexate to induce remission in early inflammatory arthritis: the EMPIRE trial. *Ann Rheum Dis* **2014**, *73*, 1027-1036, doi:10.1136/annrheumdis-2013-204882.
23. Bejarano, V.; Conaghan, P.G.; Quinn, M.A.; Saleem, B.; Emery, P. Benefits 8 years after a remission induction regime with an infliximab and methotrexate combination in early rheumatoid arthritis. *Rheumatology (Oxford)* **2010**, *49*, 1971-1974, doi:10.1093/rheumatology/keq194.
24. Durez, P.; Malghem, J.; Nzeusseu Toukap, A.; Depresseux, G.; Lauwerys, B.R.; Westhovens, R.; Luyten, F.P.; Corluy, L.; Houssiau, F.A.; Verschueren, P. Treatment of early rheumatoid arthritis: a randomized magnetic resonance imaging study comparing the effects of methotrexate alone, methotrexate in combination with infliximab, and methotrexate in combination with intravenous pulse methylprednisolone. *Arthritis Rheum* **2007**, *56*, 3919-3927, doi:10.1002/art.23055.
25. Goekoop-Ruiterman, Y.P.; de Vries-Bouwstra, J.K.; Allaart, C.F.; van Zeben, D.; Kerstens, P.J.; Hazes, J.M.; Zwinderman, A.H.; Ronday, H.K.; Han, K.H.; Westedt, M.L.; et al. Clinical and radiographic outcomes of four different treatment strategies in patients with early rheumatoid arthritis (the BeSt study):

a randomized, controlled trial. *Arthritis Rheum* **2005**, *52*, 3381-3390, doi:10.1002/art.21405.
26. Goekoop-Ruiterman, Y.P.; de Vries-Bouwstra, J.K.; Allaart, C.F.; van Zeben, D.; Kerstens, P.J.; Hazes, J.M.; Zwinderman, A.H.; Peeters, A.J.; de Jonge-Bok, J.M.; Mallée, C.; et al. Comparison of treatment strategies in early rheumatoid arthritis: a randomized trial. *Ann Intern Med* **2007**, *146*, 406-415, doi:10.7326/0003-4819-146-6-200703200-00005.
27. Goekoop-Ruiterman, Y.P.; de Vries-Bouwstra, J.K.; Allaart, C.F.; van Zeben, D.; Kerstens, P.J.; Hazes, J.M.; Zwinderman, A.H.; Ronday, H.K.; Han, K.H.; Westedt, M.L.; et al. Clinical and radiographic outcomes of four different treatment strategies in patients with early rheumatoid arthritis (the BeSt study): A randomized, controlled trial. *Arthritis Rheum* **2008**, *58*, S126-135, doi:10.1002/art.23364.
28. Markusse, I.M.; Akdemir, G.; Dirven, L.; Goekoop-Ruiterman, Y.P.; van Groenendael, J.H.; Han, K.H.; Molenaar, T.H.; Le Cessie, S.; Lems, W.F.; van der Lubbe, P.A.; et al. Long-Term Outcomes of Patients With Recent-Onset Rheumatoid Arthritis After 10 Years of Tight Controlled Treatment: A Randomized Trial. *Ann Intern Med* **2016**, *164*, 523-531, doi:10.7326/m15-0919.
29. Nam, J.L.; Villeneuve, E.; Hensor, E.M.; Conaghan, P.G.; Keen, H.I.; Buch, M.H.; Gough, A.K.; Green, M.J.; Helliwell, P.S.; Keenan, A.M.; et al. Remission induction comparing infliximab and high-dose intravenous steroid, followed by treat-to-target: a double-blind, randomised, controlled trial in new-onset, treatment-naive, rheumatoid arthritis (the IDEA study). *Ann Rheum Dis* **2014**, *73*, 75-85, doi:10.1136/annrheumdis-2013-203440.
30. Quinn, M.A.; Conaghan, P.G.; O'Connor, P.J.; Karim, Z.; Greenstein, A.; Brown, A.; Brown, C.; Fraser, A.; Jarret, S.; Emery, P. Very early treatment with infliximab in addition to methotrexate in early, poor-prognosis rheumatoid arthritis reduces magnetic resonance imaging evidence of synovitis and damage, with sustained benefit after infliximab withdrawal: results from a twelve-month randomized, double-blind, placebo-controlled trial. *Arthritis Rheum* **2005**, *52*, 27-35, doi:10.1002/art.20712.
31. Bijlsma, J.W.J.; Welsing, P.M.J.; Woodworth, T.G.; Middelink, L.M.; Pethö-Schramm, A.; Bernasconi, C.; Borm, M.E.A.; Wortel, C.H.; Ter Borg, E.J.; Jahangier, Z.N.; et al. Early rheumatoid arthritis treated with tocilizumab, methotrexate, or their combination (U-Act-Early): a multicentre, randomised, double-blind, double-dummy, strategy trial. *Lancet* **2016**, *388*, 343-355, doi:10.1016/s0140-6736(16)30363-4.
32. Verhoeven, M.M.A.; Tekstra, J.; Welsing, P.M.J.; Pethö-Schramm, A.; Borm, M.E.A.; Bruyn, G.A.W.; Bos, R.; Griep, E.N.; Klaasen, R.; van Laar, J.M.; et al. Effectiveness and safety over 3 years after the 2-year U-Act-Early trial of the strategies initiating tocilizumab and/or methotrexate. *Rheumatology (Oxford)* **2020**, *59*, 2325-2333, doi:10.1093/rheumatology/kez602.
33. Emery, P.; Bingham, C.O., 3rd; Burmester, G.R.; Bykerk, V.P.; Furst, D.E.; Mariette, X.; van der Heijde, D.; van Vollenhoven, R.; Arendt, C.; Mountian, I.; et al. Certolizumab pegol in combination with dose-optimised methotrexate in DMARD-naïve patients with early, active rheumatoid arthritis with poor prognostic factors: 1-year results from C-EARLY, a randomised, double-blind,

placebo-controlled phase III study. *Ann Rheum Dis* **2017**, *76*, 96-104, doi:10.1136/annrheumdis-2015-209057.
